# Knowledge and practice on malaria diagnosis and treatment among healthcare providers working in private health facilities in Ethiopia: A cross-sectional facility-based survey

**DOI:** 10.5281/zenodo.10870388

**Published:** 2015-07-31

**Authors:** Mesele Damte Argaw

**Affiliations:** 1 Private Health Sector Programme, P.O. Box 2372 code 1250, Addis Ababa, Ethiopia

## Abstract

**Background:**

As many as 60-80% of people in developing countries first seek malaria treatment at private rather than public health facilities, but the technical quality of private services is questionable. Before commencing a Public Private Partnership for malaria, we wanted to assess the knowledge and practice of malaria diagnosis and treatment among healthcare providers (HCPs) working in Private Health Facilities (PHFs) in Ethiopia.

**Materials and Methods:**

A facility-based cross-sectional study was conducted from April to June 2012. Quantitative and qualitative data were collected, entered and analysed using SPSS version 20. We enrolled 264 HCPs from 264 PHFs in malaria-endemic towns.

**Results:**

The majority (84.5%) of the HCPs were males, 106 (40%) were nurses and 135 (51.0%) had practiced for more than seven years. The knowledge of HCPs about the malaria programme was scored (from 1-5), and the mean was 2.52 (95% CI: 2.32-2.72), with 40.5% of the HCPs scoring above the mean. The majority knew the recommended treatment following confirmed diagnosis (91.3% for *Plasmodium vivax*, 88.6% for *P. falciparum*). 73.1% of suspected cases were investigated for parasitological diagnosis. The malaria slide positivity rate was 37.6%; however, only 60.0% of the confirmed cases were treated for malaria. Presumptive malaria treatment was offered to about 40% of patients. The adherence rate of HCPs towards prescribing the recommended first line drugs was 44.2% for chloroquine, 47.9% for ACTs and 77.9% for quinine.

**Conclusions:**

The study revealed that in Ethiopia HCPs in private practices have major gaps in knowledge and practice related to malaria case management. Therefore, provision of malaria diagnosis and case management training, supportive supervision and job aids is recommended for private healthcare providers, especially for nurses and for younger healthcare professionals.

## 1 Introduction

According to the World Health Organization malaria is still transmitted in 97 countries and territories. Globally, in the year 2013, there were an estimated 198 (range 124–238) million cases and 584,000 malaria deaths (95% uncertainty interval 367,000-755,000). Approximately 90% of these deaths occurred in the WHO Africa Region [1]. In sub-Saharan Africa, the predominant cause of malaria illness is due to infection with *Plasmodium falciparum* [1,2]. Malaria is also a major public health problem in Ethiopia, where it has consistently been reported as one of the three leading causes of morbidity and mortality in the country [2].

WHO and Roll Back Malaria recommend that malaria-endemic countries implement multiple inter-related interventions concurrently [1,3]. The major activities documented in the National Malaria Strategic Plan [4] include: community mobilization and empowerment, early diagnosis and prompt treatment, vector control, epidemic detection and mitigation, surveillance and capacity building. The key to addressing this health issue is to improve access to universal diagnosis and receiving prompt and effective treatment, which determines a positive health outcome for individuals affected by malaria [2,5]. In Ethiopia, malaria diagnosis and treatment services in all public health facilities and health posts are available free of charge. However, according to the Malaria Indicatory Surveys and National Health Account V survey reports, between one quarter and two-thirds of Ethiopians sought malaria diagnosis and treatment service from for-profit private sector facilities [6–9]. Several studies revealed that 60–80% of the population living in developing countries first seek malaria treatment at private health facilities, but that the quality of the private health services is questionable [5,8,10-12].

According to an estimate by the Federal Ministry of Health of Ethiopia, only 30% of suspected malaria cases have access to parasitological diagnosis both in public or private health facilities [2,12]. Thus, conducting a study on the knowledge and practices of healthcare providers at regular intervals may help policy makers, programme managers and healthcare providers to improve the quality of care. Moreover, this assessment might serve as baseline information to measure changes over time as a result of planned interventions to improve malaria diagnosis and treatment in the targeted facilities.

The aim of this study was to assess the perception and practice of malaria diagnosis and treatment among healthcare providers working in selected private health facilities in project-supported regions of Ethiopia.

## 2 Materials and Methods

### 2.1 Definition of key terms

#### Perception

The awareness that healthcare providers have acquired on malaria diagnosis and treatment.

#### Practice

The behaviour displayed by healthcare providers to treat patients during malaria diagnosis and treatment.

#### Supportive supervision

A range of measures to ensure that personnel carry out their activities effectively through direct, personal supervisory contact on a regular basis to guide, support and assist designated staff to become more competent in their work.

#### Private sector

The term denotes two sets of structures, i.e. the for-profit private sector, encompassing commercial enterprises of any size, and the non-profit private sector, referring to non-governmental organisations, philanthropic entities and other not-for-profit groups.

#### Public sector

Refers to national, provincial/state and district governments, municipality administrators, local government institutions, or other government and intergovernmental agencies.

#### Public-private partnerships (PPPs)

Any explicit joint programme or project involving public and private collaboration to provide services. These include contracting between the public sector (either governments or development agencies) and private providers to provide goods and services [11].

### 2.2 Study area and design

**Figure 1.**
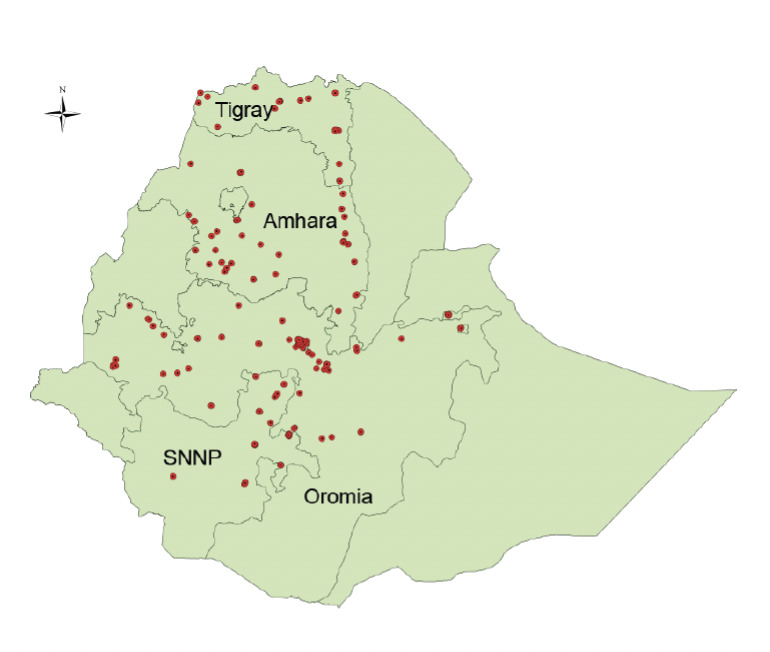
Map of Ethiopia showing the four Regions where the study took place. Red dots indicate surveyed facilities (Map: PHSP supported malaria sites, 2013).

An institution-based cross-sectional quantitative and qualitative study was conducted from April to June 2012. Ethiopia is located northeast of Africa [13] and has an area of 1.1 million km^2^ [2]. Nearly 75% of the land mass is endemic for malaria, where approximately 52 million people reside (68% of the total population) [2,4]. In Ethiopia, there are approximately equal numbers of public and private health facilities, i.e. 100 public hospitals, 95 privately owned hospitals, 1,365 public health centres, and 2,853 private higher- and medium-level clinics, of which 570 are considered higher-level clinics [2,14]. This study was conducted in large and highly malaria-endemic states (Amhara; Oromia; Southern Nations, Nationalities and Peoples [SNNP]; and Tigray Regions; Figure 1); 80.7% of 2,627,182 malaria cases from across Ethiopia were reported from these selected regional states [14].

### 2.3 Sampling and sample size

The Four Regional States (Amhara, Oromia, Tigray and SNNP) were selected using purposive sampling technique, as they are targeted for a Public Private Partnership for malaria care services by the Private Health Sector Programme. All facilities located in 36 administrative zones which were accessible with a field vehicle were selected for this study. A total of 264 private health facilities and 264 index healthcare providers were enrolled, of which 77 were from Amhara, 51 were from Tigray, 48 were form SNNP and 88 were from Oromia. However, due to time and cost constraints, patient data were collected from six lower clinics, 25 medium clinics, eight higher clinics and one hospital. Per institution, one healthcare worker, who was responsible for diagnosis and treating patients on the day of the survey, participated.

### 2.4 Data collection

Quantitative data were collected using modified tools used in a previous study [12]. Data extraction tools were developed by the researcher and captured qualitative data. Consistency of the tools (i.e. questionnaires, a checklist and data extraction formats) was checked by different public health professionals. Enumerators collected the data through interview, direct observation and record review. The questionnaires contained socio-demographic information, knowledge, practice, job aids and supportive supervision-related questions.

### 2.5 Data analysis

Data were entered into Microsoft Office Excel 2010, cleaned and analysed using SPSS version 20.0. To identify statistical significant variables, a chi-square test with P=0.05 significance levels was employed.

### 2.6 Ethical considerations

Permission was granted from the Private Health Sector Programme and Regional Health Bureaus. The standard consent form recommended by the Ethiopian Science and Technology Commission (ESTC) for health research indicated in the National Health Research Ethics Review Guidelines of Ethiopia [15] was adopted. Verbal consent was obtained from owners of the facility and participants. Participants were informed about the issue of confidentiality and that they had the full right to refuse or discontinue participating in the research without any compromise in the relationship with the owners of the private health facilities; hence, the study subjects participated voluntarily in the study.

## 3 Results

264 Private health facilities enrolled, of which 48 (18.2%) were from SNNP, 51 (19.3%) were from the Tigray Region, 77 (29.2%) from the Amhara Region and 88 (33.3%) from the Oromia Region. The level of the health facilities that participated in this study was: 12 (4.5%) Hospital, 97 (36.7%) Higher Clinics, 133 (50.4%) Medium Clinics and 22 (8.3%) Lower Clinics. The majority (253; 95.9%) of these facilities are located in urban areas. A little more than one-third of the surveyed facilities (99; 37.5%) were operational for 6+ years, whereas half (138; 52.3%) had been serving communities 2-5 years; only 27 (10.2%) of the surveyed facilities had been operational ≤1 year. Almost all private facilities (240; 90.0%) were for-profit, 14 (5.3%) were not-for-profit and 10 (38.0%) were workplace clinics (Table 1).

**Table 1.** Characteristics of the private health facilities surveyed in Ethiopia, April-June 2012.

Variable	Level of health facility				
	Hospital	Level Higher Clinic	of health facility Medium Clinic	Lower clinic	Total (%)
**Number of facilities surveyed by Region**					
Tigray	0	14	18	19	51 (19.3%)
Amhara	4	20	53	0	77 (29.2%)
SNNP	2	13	30	3	48 (18.2%)
Oromia	6	50	32	0	88 (33.3%)
Total (%)	12 (4.5%)	97 (36.7%)	133 (50.4%)	22 (8.3%)	264 (100%)
**Years of service in the study area**					
≤ 1	0	11	16	0	27 (10.2%)
2-5	10	47	68	13	138 (52.3%)
≥ 6	2	39	49	9	99 (37.5%)
**Ownership (Private)**					
For-profit	11	82	126	21	240 (90.9%)
Not-for-profit	1	10	3	0	14 (5.3%)
Workplace	0	5	4	1	10 (3.8%)
**Location**					
Rural	0	6	4	1	11 (4.1%)
Urban	12	91	129	21	253 (95.9%)

In the selected private health facilities, 264 healthcare providers, who were directly engaged in malaria case management, were interviewed. The majority of the respondents (223; 84.5%) were males. The mean age of the participants was 35.21 (95% CI; 34.2–36.2) and the median age was 34 years. The age of participants ranged from 20 to 67 years. One hundred twenty two (46.2%) respondents were found in the age category 25–34 years, followed by 43 (31.1%) in the age category 35–44 years. The majority of the respondents (106; 40.2%) were nurses, followed by 67 (25.4%) health officers. Two-thirds of the respondents (180; 68.0%) had been employed for less than ≤ 3 years in the surveyed private health facilities. The mean number of years of services tenured in the surveyed facility was 2.8 (95% CI; 2.57–3.06). However, half of the respondents (135; 51.0%) had been employed in professional services within the Ethiopian health system for ≥ 7 years. The mean length of professional services tenured in the health system by the respondents was 9.88 (95%CI; 8.9–10.8) years (Table 2).

**Table 2.** Characteristics of healthcare providers in surveyed private health facilities.

Variable	Type of health facility				
	Hospital	Higher Clinic	Medium Clinic	Lower Clinic	Total (%)
**Gender**					
Male	12	73	117	21	223 (84.5)
Female	0	24	16	1	41 (15.5)
**Age group**					
20-24 years	0	9	4	4	17 (6.4)
25-34 years	8	40	71	3	122 (46.2)
35-44 years	2	35	35	10	82 (31.1)
≥ 45 years	2	13	23	5	43 (16.3)
**Profession**					
Specialist, Medical Doctor	8	18	3	0	29 (11.0)
General Practitioner	3	23	2	0	48 (18.2)
Health Officer	0	5	60	2	67 (25.4)
Nurse	1	50	42	13	106 (40.2)
Junior Nurse/Health Assistant	0	0	2	7	9 (3.4)
Other	0	1	4	0	5 (1.9)
**Years of service**					
≤ 3 years	9	68	91	12	180 (68.0)
4-6 years	3	25	35	6	69 (26.0)
≥ 7 years	0	4	7	4	15 (6.0)
**Years of professional service**					
≤ 3 years	0	15	22	3	40 (15.0)
4-6 years	8	30	47	4	89 (34.0)
≥ 7 years	4	52	64	15	135 (51.0)

### 3.1 Knowledge of healthcare providers

Only very few providers (21; 8.0%) had ever participated in malaria case management trainings. Two-thirds (183; 69.3%) of the respondents had ever seen a malaria rapid diagnostic test (RDT). However, out of these, only 101 (55.2%) reported that they have the knowledge to perform the test and were able to read the test results. Healthcare providers were asked to list the national malaria prevention and control strategies, and most (213; 80.0%) correctly named selective vector control (indoor residual spraying/IRS, long-lasting insecticide-treated nets/LLINs and environmental management) measures. 157 (59.5%) Identified early case diagnosis and prompt treatment. However, more than half of the healthcare providers could not name the other three strategies. The majority of the respondents (234; 88.6%) and 241 (91.3%) of healthcare providers knew the national recommended first-line treatment for confirmed *P. falciparum* and *P. vivax* malaria, respectively. A little more than one-third (96; 36.4%) of the healthcare providers correctly knew the first-line treatment that follows clinical presumptive malaria diagnosis (Table 3).

**Table 3.** Perception of healthcare providers on malaria prevention and control in Ethiopia, April-June 2012.

Characteristic	Affirmative (%)
Attended malaria case management training	21 (8.0)
Ever seen an RDT cassette	183 (69.3)
Have the capacity to perform test and read the result correctly (n=183)	101 (55.2)
**National malaria control strategies**	
Community mobilization and empowerment	150 (56.8)
Selective vector Control (IRS, LLINs, Environmental management)	213 (80.7)
Early diagnosis and prompt treatment	157 (59.5)
Surveillance, epidemic preparedness and mitigation	76 (28.8)
Capacity Building (training of HWs, HMIS)	69 (26.2)
**Recommended treatment**	
Presumed malaria: ACT	96 (36.4)
Confirmed *P. falciparum* malaria: ACT	234 (88.6)
Confirmed *P. vivax* malaria: Chloroquine	241 (91.3)
Malaria in first trimester of pregnancy & infant <5 kg: Quinine tablet	180 (68.2)
Severe and complicated malaria: Artesunate/Quinine	224 (84.8)

**Figure 2.**
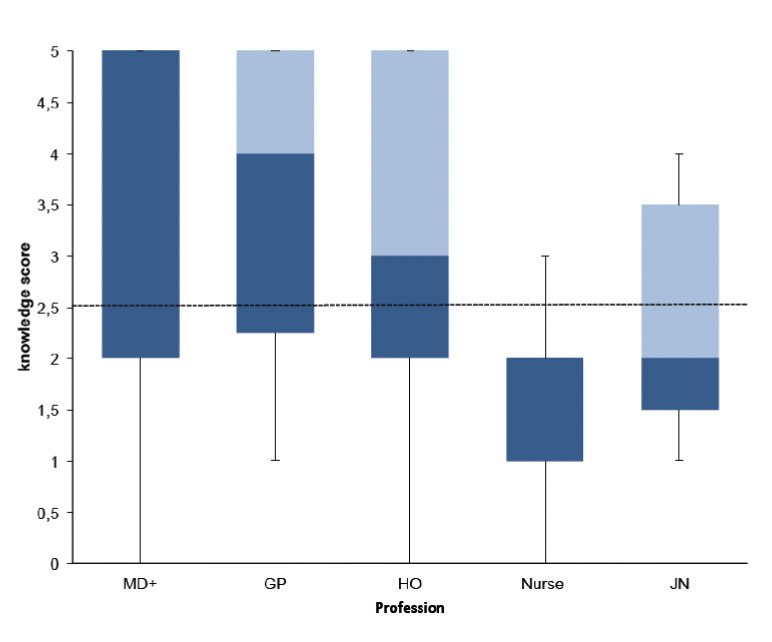
Boxplot showing knowledge scores according to profession for healthcare providers in the study. The graph depicts the maximum, minimum, median and interquartile ranges. The dotted line indicates the average score. MD+ = Specialists, Medical Doctors, GP = General Practitioner, HO = Health Officer, JN= Junior Nurse.

The mean knowledge score on the national malaria prevention and control strategies were computed out of five. The overall mean score with 95% CI was 2.52 (2.32–2.72). The highest score was recorded by General Practitioners with a mean score of 3.52 (95% CI; 3.07–3.96), followed by Specialists, Medical Doctors, with a mean score of 3.48 (95%CI; 2.68–4.28), followed by Health Officers with a mean score of 2.91 (95% CI; 2.53–3.28). The fourth-highest score was recorded by junior nurses or health assistants with a mean of 2.44 (95% CI; 1.57–3.31), and the lowest score was recorded by nurses with a mean score of 1.63 (95% CI; 1.43–1.82). Overall, 40.5% of the respondents reached a knowledge score that was greater or equal to the mean score (Figure 2).

**Table 4.** Practices of healthcare providers regarding malaria case management in 40 private health facilities in Ethiopia, April-June 2012.

Characteristic	Frequency	%
All outpatient cases	104,904	*NA
Malaria-suspected cases	25,311	24.1
Malaria cases diagnosed and treated	11,466	10.9
Male	6021	52.5
Female	5445	47.5
Pregnant mother	66	0.6
Blood film tested	18,496	73.1
Confirmed malaria cases	6953	37.6
*Plasmodium falciparum*	3475	50.0
*Plasmodium vivax*	3134	45.1
Mixed (*Pf* and *Pv*)	344	4.9
Presumptive malaria diagnosis	4513	39.4
All causes of admission	4918	NA
Malaria admission	300	6.1
All causes of death	12	NA
Malaria-related deaths	2	16.7

*NA, not applicable

### 3.2 Diagnosis and treatment

Nearly all (244; 92.0%) of the respondents reported that they used parasitological diagnosis with microscopy, 88 (33.0%) diagnosed malaria on clinical/presumptive basis and 40 (15.0%) used RDTs for parasitological confirmation of malaria. According to the data collected from the morbidity and mortality registers of 40 selected private health facilities, there were 104,940 patients registered during the two-month study period. Out of these, 25,311 (24.1%) were suspected cases and 11,466 (10.9%) were diagnosed and treated as malaria patients. Sixty-six (0.6%) of the cases were pregnant mothers. Blood film was performed for 18,496 (73.1%) of the suspected cases and parasitological confirmation obtained for 6953 (37.6%) cases. Of these, 3475 (50.0%) were *P. falciparum* infections, 3134 (45.1%) *P. vivax*, and the remaining 344 (4.9%) were found with mixed *Plasmodium* infections. A little more than one-third of malaria patients (4513; 39.4%) were diagnosed based on a clinical/presumptive approach. The malaria admission rate was 300 (6.1%) and death due to malaria was 2 (16.7%) (Table 4).

The distribution of diagnosed malaria cases against age category is very important to identify high-risk groups. The total number of malaria cases reported was 11,466. Of these, 268 (2.3%) were infants, 1776 (15.5%) were children aged 1-4 years, 1768 (15.4%) were children aged 5-14 years and the remaining 7614 (66.4%) were adults aged fifteen or above (Table 5). One-fifth of the malaria cases reported were in children under five.

**Table 5.** Distribution of malaria across age groups based on records from 40 private health facilities in Amhara and SNNP Regions, April-June 2012.

Region	Months	≤1 yr (%)	1-4 yrs (%)	5-14 yrs (%)	≥15 yrs (%)	Total
Amhara	2	149 (3.4)	414 (10.4)	540 (13.5)	2848 (71.3)	3993
SNNP	2	119 (1.6)	1362 (18.2)	1228 (16.4)	4766 (63.8)	7473
Total	2	268 (2.3)	1776 (15.5)	1768 (15.4)	7614 (66.4)	11466

**Figure 3.**
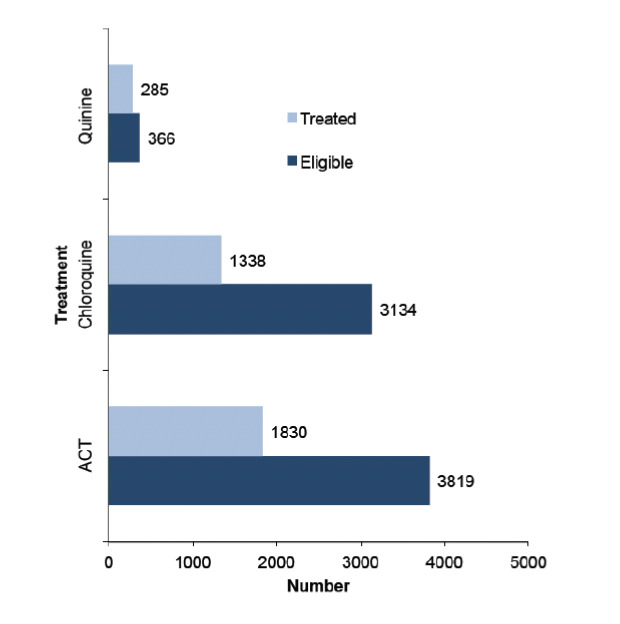
Practice of private healthcare providers in adhering to national recommended guidelines for malaria treatment. The graph compares eligible groups for each drug against the actual practice of healthcare providers.

Half of the patients (1830; 47.9%) who were eligible for recommended treatment with artemether-lumefantrine (AL) were correctly treated. Similarly, a little less than half of the patients (1386; 44.2%) who were eligible for chloroquine had been treated appropriately. Moreover, 28 (77.9%) of patients eligible for artesunate injection/ quinine tablets had received the desired prescription (Figure 3).

### 3.3 Patient-health worker communication

In most cases (256 (97%)), health information was provided to patients or caretakers. Private health providers reported that the topics they addressed in the management of malaria cases included: the mode of prevention (217 (84%)), the importance of compliance to prescribed drugs (181 (70.7%)) and the importance of returning back if conditions worsened or fever persisted for 72 hours (126 (49.4%)) (Table 6).

**Table 6.** Malaria-related health inform provision by healthcare providers in Ethiopia, April-June 2012.

Characteristics	Affirmative (%)
Advice client/patients malaria-related health information	256 (97.0)
If yes, on what topics/issues (n=256) Mode of transmission	111 (43.4)
Mode of prevention	217 (84.8)
Importance of early diagnosis and prompt treatment	111 (43.4)
Importance of compliance with treatment	181 (70.7)
Importance of returning back if condition worsens or fever persists	126 (49.4)

### 3.4 Supportive supervision

Nearly two-thirds (170; 64.4%) of healthcare providers reported that they received supportive supervision regarding malaria prevention and control. The supervisors were categorised into three main groups of players, of which 22.35% were senior internal staff, 22.9% were experts of the Regional Health Bureau and 5.7% were experts from the World Health Organization’s office.

### 3.5 Determinant factors

Relationships between socio-demographic characteristics and mean knowledge scores were cross-tabulated to determine differences, using a chi-square (*Χ*^2^) test. The mean score of male respondents was higher than their female counterparts (*Χ*^2^=5.25, P<0.01). The younger age group scored significantly lower than older age groups (*Χ*^2^=6.74, P=0.009). The profession of healthcare providers had a direct, statistically significant relationship with their knowledge scores. Taking nurse as a reference profession, the *Χ*^2^ test results for Specialist, General practitioner and Health Officer were 33.74, 57.54 and 28.86, respectively. Professional services tenured were significantly positively associated with higher levels of achievement in knowledge scores. The *Χ*^2^ value for the first three years of service tenured computed against seven or more years of service was 15.16 (P<0.001). The *Χ*^2^ value for 4-6 years of service year tenured against 7 or more was 0.018 (P=0.67) (Table 7).

**Table 7.** Relationships between various socio-demographic parameters and knowledge scores regarding national malaria prevention and control strategies in Ethiopia, April-June 2012.

Variable	Above mean score	Below mean score	X^2^	P-value
**Gender**				
Male	97	126	5.25	<0.01
Female	10	31		
**Age category**				
20-34 years	46	93	6.74	0.009
35-67 years	61	64		
**Profession**				
Specialist, MD	19	10	33.74	<0.001
General Practitioner	36	12	57.54	<0.001
Health Officer	34	33	28.86	<0.001
Nurse	14	92		
**Service tenured**				
≤3 years	5	35	15.16	<0.001
4-6 years	39	50	0.18	0.67
≥7 years	63	72		

## 4 Discussion

Ethiopia has adopted a policy to identify all suspected malaria cases within 24 hours after the occurrence of signs or symptoms, perform parasitological diagnosis for all suspected malaria cases and treat all confirmed cases with nationally recommended, effective antimalarial drugs. Malaria services are free of charge in public facilities and at community level through a health extension programme. Its purpose is to improve access for the poor and vulnerable segments of the community [2]. However, a significant segment of the Ethiopian population continue to receive care through the private health sector [6-9]. Furthermore, Ethiopia is aiming beyond sustained malaria control to the elimination of malaria in selected geographic areas, which demands the involvement of several stakeholders, including private healthcare providers [2,4]. This study investigated the knowledge and practice of private health providers on malaria case management.

The perception of private healthcare providers was estimated through their correct knowledge of the recommended national malaria case management strategy. The level of favourable perception was 40.5%, which was measured by the knowledge score of participants greater than or equal to the average score among the study participants. This study revealed that private healthcare providers knew two out of five components of the national malaria prevention and control strategies. The majority (80.7% and 59.5%, respectively) of the healthcare providers cited selective vector control and early diagnosis/prompt treatment. In line with this, a study conducted in Angola also documented the need to address these knowledge gaps among healthcare providers [16].

Private healthcare providers had a high level of knowledge on the recommended drugs for each species of *Plasmodium*. The majority (91.3%, 88.6% and 84.8%, respectively) had knowledge of the nationally recommended drugs against *P. vivax*, *P. falciparum* and for malaria infection during the first trimester of pregnancy. However, only 36.4% knew the correct recommended drug for presumed diagnosed malaria. There was a statistically significant difference among younger staff, female respondents, nurses and the number of years of professional service (*P*<0.05), with knowledge scores computed against their counterparts. A previous study conducted in Oromia Regional state identified the knowledge gaps to manage malaria cases properly and recommended to consider inclusion of private healthcare providers in the national malaria control programme [12].

Only 8% of the study participants had attended malaria case management training. Similar findings were reported by Jerene *et al.* [12], in which 12 (4%) of private health sector providers had attended any kind of training in the previous year. This finding was much lower than the 43% of health providers that had participated in on-the-job training in Uganda [17]. This could be due to the fact that the public sector might have a stronger concern about the quality of malaria prevention and control services than private providers [18]. Moreover, there is very limited experience of working towards a shared goal within the principles of public-private partnerships [11].

Assuming that the presence of RDT for malaria diagnosis is safe and effective in promoting confirmed case management [19], we asked participants for their experiences regarding this method. Over two-thirds of the study participants (69.3%) had ever seen an RDT cassette for malaria diagnosis. However, only half of them had sufficient confidence in performing the test and interpret results correctly. This could be due to the fact that the value and demand of the beneficiaries might encourage private sector providers to accept new technology much faster than the public sector. Furthermore, the logistics management system of the private sector might be easier and more functional than the in public sector [11].

Almost all (97.0%) of healthcare providers reported that they provided malaria-related health messages for patients or care takers. This finding is somewhat higher than that in the study by Jerene *et al.* [12], in which 81% of healthcare providers had advised patients on malaria prevention and control mechanisms in ten zones of Oromia regional state of Ethiopia. This could be due to a limited number of malaria cases reported by dedicated private providers to maintain better reputations [11].

Assuming that most clients prefer private healthcare providers for available diagnostic services, the Comprehensive Lab registers were reviewed. Of all malaria-suspected cases, 73.1% were investigated for laboratory-based parasitological confirmation for malaria. This finding was much higher than the 30.7% malaria-suspected cases in Angola documented by Rowe *et al.* [16]. The discrepancies may be explained by differences in time, patient load and level of health facilities. And unlike this study, which enrolled private for-profit facilities, Rowe *et al.* [16] enrolled public facilities, i.e. 5 hospitals and 28 health centres [16]. As most of the private facilities are equipped with a single lab technician, there was the possibility to diagnose malaria based on clinical signs and symptoms. Therefore, 5.2% of private health facilities used RDTs to confirm malaria. A similar finding was reported in a study conducted in Oromia region, where 12 facilities were using RDTs to diagnose malaria [12]. RDTs can be performed by any healthcare provider with basic training. Healthcare providers might use it either during duty house and/or case managers might prefer to use RDTs rather than microscopy for more reliable results.

In two months, 24.1% of all patients visited private health facilities for malaria diagnosis and treatment services. This is in line with the previously reported 25% of mothers visiting private health facilities with their febrile child [8]. Although all suspected malaria cases were expected to undergo confirmatory tests, only 73.1% of these patients were being sent to lab facilities to confirm diagnosis. This was much higher than the 30.7% of suspected malaria cases tested in Angola [16]. Only 60.6% of malaria patients had lab-confirmed results, whereas the remaining 39.4% were treated based on a presumptive diagnosis. Among these patients, 44.3-77.9% were treated with the correct prescription of artemether-lumefantrine, chloroquine or quinine when required. This finding was in line with previous studies in which approximately one half of malaria patients received correct treatment [16,20,21]. A study carried out on routine practices by Zurovac *et al.* [22] revealed that 30-87% of patients were unnecessarily treated with antimalarial drugs.

There were only very few job aids and patient information brochures. The most frequently observed job aid was a drug-dosing schedule. Other very important job aids, such as the national malaria guidelines, diagnosis and treatment procedures or algorithms were almost nowhere available. This finding was in line with a study in Oromia, where only 10% of private health facilities had malaria guidelines [12]. Printing job aids and national guidelines is not affordable by individual private health facilities. Therefore, public facilities and other stakeholders ought to supply these essential materials to ensure standard care services both in public and private facilities.

One-to-one technical support and supportive supervision have positive outcomes through solving challenges [12,19,22,23]. Two-thirds (64.4%) of healthcare providers reported that they had received supportive supervision form internal senior staff, regional health bureau staff or World Health Organization Offices. Although the technical support given to healthcare providers was merely related to regulatory control, there was a little technical support in Ethiopia and Uganda [12,17]. Future studies are recommended after planned interventions to measure the quality of outpatient malaria care management in the formal private health sector.

### Limitations

Private health facilities are expected to prescribe patients with the recommended anti-malarials and refer malaria cases to the nearest drug outlets; this study did not consider the duration of stock out of essential anti-malaria supplies as a major factor for desired quality of malaria service in the private health facilities. The malaria morbidity and case management information was extracted from 40 facilities, which might make it difficult to generalise the findings for selected private health facilities.

## 5 Conclusions

This study revealed that healthcare providers working in private practices in Ethiopia appear to have major gaps in knowledge and practice related to correct malaria case management. Therefore, the provision of malaria diagnosis, case management training, supportive supervision and job aids is highly recommended for private health care providers, especially coaching nurses and younger health professionals on current national malaria prevention and control programme policies.
